# The organization of domains in proteins obeys Menzerath-Altmann’s law of language

**DOI:** 10.1186/s12918-015-0192-9

**Published:** 2015-08-11

**Authors:** Khuram Shahzad, Jay E. Mittenthal, Gustavo Caetano-Anollés

**Affiliations:** Illinois Informatics Institute, Urbana, IL 61801 USA; Department of Cell and Developmental Biology, Urbana, IL 61801 USA; Department of Crop Sciences, Evolutionary Bioinformatics Laboratory, University of Illinois, 332 NSRC, Urbana, IL 61801 USA

## Abstract

**Background:**

The combination of domains in multidomain proteins enhances their function and structure but lengthens the molecules and increases their cost at cellular level.

**Methods:**

The dependence of domain length on the number of domains a protein holds was surveyed for a set of 60 proteomes representing free-living organisms from all kingdoms of life. Distributions were fitted using non-linear functions and fitted parameters interpreted with a formulation of decreasing returns.

**Results:**

We find that domain length decreases with increasing number of domains in proteins, following the Menzerath-Altmann (MA) law of language. Highly significant negative correlations exist for the set of proteomes examined. Mathematically, the MA law expresses as a power law relationship that unfolds when molecular persistence *P* is a function of domain accretion. *P* holds two terms, one reflecting the matter-energy cost of adding domains and extending their length, the other reflecting how domain length and number impinges on information and biophysics. The pattern of diminishing returns can therefore be explained as a frustrated interplay between the strategies of economy, flexibility and robustness, matching previously observed trade-offs in the domain makeup of proteomes. Proteomes of Archaea, Fungi and to a lesser degree Plants show the largest push towards molecular economy, each at their own economic stratum. Fungi increase domain size in single domain proteins while reinforcing the pattern of diminishing returns. In contrast, Metazoa, and to lesser degrees Protista and Bacteria, relax economy. Metazoa achieves maximum flexibility and robustness by harboring compact molecules and complex domain organization, offering a new functional vocabulary for molecular biology.

**Conclusions:**

The tendency of parts to decrease their size when systems enlarge is universal for language and music, and now for parts of macromolecules, extending the MA law to natural systems.

## Background

*“Life is a relationship between molecules, not a property of any one molecule”* Emile Zuckerkandl and Linus Pauling [1]

Early last century, Paul Menzerath proposed a generality for language constructs [2]. He found that longer syllables contained shorter articulated sounds and later revealed that words with more syllables were phonetically shorter. He summarized his findings with the motto: “*the greater the whole, the smaller its constituents*” (*“Je größer das Ganze, desto kleiner die Teile”*) [3]. These qualitative statements were later elaborated mathematically by Gabriel Altmann [4] and supported by statistical analyses of many languages and linguistic and phonetic relationships of many types. One general formulation of the accepted Menzerath-Altmann’s (MA) law that adds the effect of hierarchy in the makeup of parts [4] follows eq. (1)

1 $$ y(x)=A{x}^b{e}^{-cx} $$

with *y(x)* being the length of the parts, *x* representing the length of the system (or constructs of parts), and *A*, *b* and *c* fitting parameters. *x* can also represent a discrete variable describing the number of parts that make up the system. A more general formulation adds dependences on additional variables [5]. *y(x)* is generally measured by counting parts defined at a deeper level of the system’s organization (e.g., amino acids of domains). This general formulation of the law accommodates the effects of multi-level structure that is typical of language. Two special cases of the equation occur when *b* = 0 or *c* = 0. The first mathematical formulation describes how the length or size of parts *y(x)* decreases monotonically with the length or size of systems. However, the second formulation, eq. (2)

2 $$ y(x)=A{x}^b $$

is the most commonly used equation of the MA law, since it enables computation of fitting parameters in log-log plots. This equation delimits a curve of a general two-parameter power law form.

Language-like behavior has been extended to music [6] and recently to genomes [7–10], making the MA law a generality of both natural and human-made systems. In biology, Menzerath’s tendency of the mean size of the parts to decrease as the number of parts increases in a system was shown to be expressed at the cellular and biomolecular level as negative correlations between the mean chromosome length and the number of chromosomes or the size of genomes [7, 8] and mean exon size and the number of exons [9]. Very recently, quantitative linguistic distribution models and statistical analyses have also been used to explore the self-organization of coding and non-coding genomic components [11] and amino acid length distributions of proteins [12]. Here we report that the organization of structural domains in proteins obeys the MA law at the proteome level.

Protein molecules are eminently modular [13]. Recurrent substructures appear in different molecular contexts. This is particularly evident when considering the structural domains of proteins. Domains are 3-dimensional (3D) atomic arrangements of elements of secondary structure that fold into well-packed structural units [14, 15] and are evolutionarily conserved [16–18]. They fold and function largely independently and contribute to overall protein stability by establishing a multiplicity of intramolecular interactions [19]. In evolution, domains combine in multidomain proteins by fusion or excise by fission processes, driven mostly by the forces of genome rearrangement [20]. Consequently, the resultant ‘architectures’ afford functional diversity drawn from both domain structure and domain organization [21]. This fact is made evident by wide co-option of ancient enzymatic activities in metabolic networks [22]. The dynamics of the complex evolutionary mechanics of domain combination results in global patterns of domain gain and loss that materialize differently in the proteomes of the three superkingdoms of life, Archaea, Bacteria and Eukarya [23]. Moreover, phylogenomic analyses of protein domain structures in hundreds of proteomes have shown that the bulk of multidomain proteins appeared explosively quite late in evolution [20]. The rise of domain organization possibly impacted constraints imposed on early proteins by folding speed and protein flexibility [24]. Domain combinations also affected the length of domains and proteins [25, 26], with younger domains exhibiting simpler and smaller structures [27].

Multidomain proteins, which globally make a significant minority (26–32 %) of proteins in proteomes (they are highly represented in eukaryotes), have on average substantially smaller domains than single domain proteins [25]. This trend persists despite proteins of bacterial and archaeal microbes evolving reductively relative to those of eukaryotes by significant shortening of non-domain linker sequences that do not affect domain length. Here we explore how the number of domains in proteins impacts the length of domains. Using a selected set of proteomes sampled from the three superkingdoms we dissect significant law-abiding reductive patterns operating at the proteome level. Our results uncover the important role of cellular economy, as it imposes strong evolutionary pressure on domain structure and organization and biases trade-off relationships needed for organismal persistence.

## Results and discussion

### The longer the protein the smaller its structural domains

**Fig. 1:**
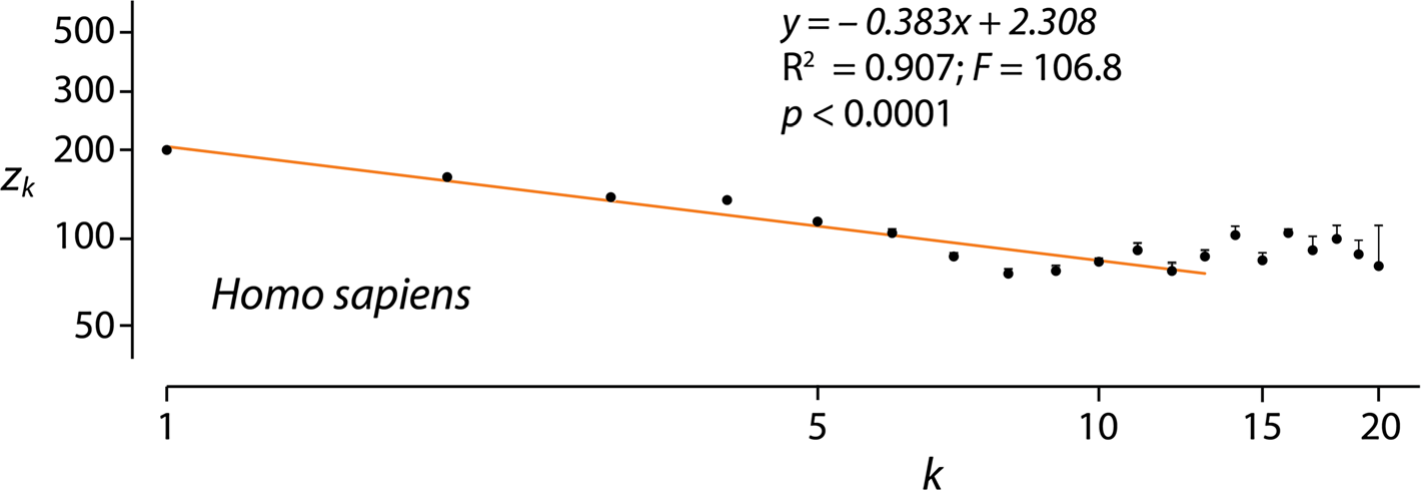
A log-log plot describing how average structural domain length (*z*_*k*_) of a protein decreases with the number of protein domains it holds (*k*) for proteins in the proteome of *Homo sapiens*. Circles show the mean values of average number of domain lengths within a *k* value. The horizontal bars on the circles depict the standard error of the means. The red line indicates the linear fitting (regression) line, which does not pass through points with *k* > *K’*

We studied the dependence of the average domain length (*z*_*k*_) of a protein on the number of protein domains it holds (*k*) for a set of 60 proteomes representing organisms in superkingdoms Archaea and Bacteria and kingdoms Metazoa, Fungi, Plants and Protista of superkingdom Eukarya. Each and every one of the 60 proteomes examined showed a significant negative correlation between average domain lengths and numbers of domains in proteins, both in logarithmic scale, when using a weighted nonlinear least-squares curve fitting approach (Table 1). To avoid fitting artifacts due to a small minority of proteins harboring high number of domains, we excluded the terminal outliers while retaining an average of 99.44 % (±0.91 SD) (range 96.8–100 %) of entries. Figure 1 shows an example plot describing tight correlation in the proteomic data of *Homo sapiens*. The linear regression lines in the log-log plots showed high coefficients of determination (R^2^) with values ranging 0.85–1.00 and significant *F* test-derived correlations (*F* test; *F* = 11.5-2714; *p* < 0.0001-0.133; only 3 proteomes had *p*-values higher than 0.05) (Table 1). Since R^2^ > 0.85 values are assumed to indicate satisfying fits and *F*-test outliers may result from methodological weaknesses of the regression statistics [27], both statistics support in concert significant goodness of the regression fits over ranges of *k*. In all cases, domain length decreased monotonically with number of domains in proteins, delimiting a MA law for proteomes. Slopes (*b*) in the log-log plots ranged −0.113 to −0.404 (Table 1), making explicit the negative correlation typical of the MA power law.

**Table 1:** Summary table of correlation data for the 60 proteomes examined

No	Kingdom	Genus/Species	G.a.	Total proteins	Selected proteins	% data selected	Slope (*b*) (± SE)	Intercept (*A*) (± SE)	R^2^	Genome size (kb)	L*	*L*_*e*_	*F*-value	*p*-value
1	Metazoa	*Homo sapiens*	hs	30610	30516	99.69	–0.354 (± 0.055)	199.555 (± 14.935)	0.91	3080436	522	286	106.83	<0.0001
2	Metazoa	*Apis mellifera*	ai	15858	15708	99.05	–0.308 (± 0.061)	212.979 (± 16.299)	0.91	200000	467	281	85.95	<0.0001
3	Metazoa	*Branchiostoma floridae*	bf	33445	33346	99.7	–0.404 (± 0.075)	197.516 (± 21.842)	0.91	480405	505	267	181.82	<0.0001
4	Metazoa	*Caenorhabditis elegans*	cl	14297	14224	99.49	–0.351 (± 0.037)	224.737 (± 6.234)	0.93	100272	530	286	116.85	<0.0001
5	Metazoa	*Danio rerio*	da	23072	22978	99.59	–0.374 (± 0.071)	206.682 (± 17.610)	0.92	1700000	504	285	147.17	<0.0001
6	Metazoa	*Gallus gallus*	gg	14376	14302	99.49	–0.304 (± 0.027)	203.088 (± 6.813)	0.95	1000000	573	295	251.53	<0.0001
7	Metazoa	*Lottia gigantea*	gy	12223	12162	99.5	–0.345 (± 0.087)	198.757 (± 20.942)	0.93	359500	441	253	143.64	<0.0001
8	Metazoa	*Ciona intestinalis*	is	11913	11773	98.82	–0.336 (± 0.051)	215.482 (± 12.309)	0.92	116700	497	285	78.86	<0.0001
9	Metazoa	*Xenopus laevis*	xl	23167	23151	99.93	–0.324 (± 0.020)	196.487 (± 4.213)	0.9	205432	456	262	100.49	<0.0001
10	Metazoa	*Daphnia pulex*	d7	11750	11705	99.62	–0.252 (± 0.045)	191.214 (± 9.103)	0.92	197300	437	242	100.8	<0.0001
11	Plants	*Arabidopsis thaliana*	at	15858	15856	99.99	–0.256 (± 0.067)	215.928 (± 12.090)	0.92	119707	470	271	68.11	0.0002
12	Plants	*Carica papaya*	r6	12095	12091	99.97	–0.149 (± 0.030)	190.871 (± 1.098)	0.9	271733	401	236	36.23	0.0038
13	Plants	*Chlamydomonas reinhardtii*	cy	7132	7073	99.17	–0.156 (± 0.059)	192.702 (± 8.648)	0.89	100000	581	234	16.59	0.0553
14	Plants	*Chlorella* sp	h2	6153	6147	99.9	–0.205 (± 0.034)	200.449 (± 5.810)	0.9	40000	473	248	45.33	0.0011
15	Plants	*Cyanidioschyzon merolae*	ya	3152	3127	99.21	–0.255 (± 0.041)	225.731 (± 7.531)	0.99	16520	525	281	158.93	0.0062
16	Plants	*Medicago truncatula*	mw	15858	14899	93.95	–0.045 (± 0.018)	183.279 (± 2.804)	0.97	500000	410	225	103.26	0.002
17	Plants	*Oryza sativa*	os	15858	15773	99.46	–0.121 (± 0.056)	206.214 (± 9.984)	0.85	420000	579	284	11.46	0.0773
18	Plants	*Physcomitrella patens*	pw	13310	13280	99.77	–0.178 (± 0.065)	205.616 (± 10.894)	0.93	453929	441	261	38.61	0.0084
19	Plants	*Vitis vinifera*	vt	17268	17241	99.84	–0.124 (± 0.035)	210.018 (± 5.922)	0.93	504600	461	274	38.68	0.0084
20	Plants	*Populus trichocarpa*	pt	15858	15857	99.99	–0.113 (± 0.027)	194.256 (± 1.770)	0.83	550000	454	244	24.91	0.0041
21	Fungi	*Ashbya gossypii*	go	2908	2897	99.62	–0.257 (± 0.061)	233.156 (± 14.176)	0.98	9200	532	293	136.05	0.0014
22	Fungi	*Candida glabrata*	gl	3155	3143	99.62	–0.267 (± 0.094)	235.165 (± 20.535)	0.92	12280	548	296	34.59	0.0098
23	Fungi	*Kluyveromyces waltii*	kw	3106	3094	99.61	–0.257 (± 0.153)	230.109 (± 28.805)	0.93	11000	509	286	37.22	0.0088
24	Fungi	*Laccaria bicolor*	lo	7148	7133	99.79	–0.164 (± 0.040)	208.118 (± 7.009)	0.95	58683	469	255	52.15	0.0055
25	Fungi	*Neurospora crassa*	ns	4745	4723	99.54	–0.271 (± 0.126)	239.997 (± 26.390)	0.93	37097	586	297	38.55	0.0084
26	Fungi	*Saccharomyces cerevisiae*	xs	3517	3503	99.6	–0.251 (± 0.065)	233.237 (± 13.702)	0.93	12069	556	295	41.92	0.0075
27	Fungi	*Aspergillus nidulans*	an	6335	6255	98.74	–0.288 (± 0.153)	247.290 (± 30.285)	0.93	30166	542	300	25.92	0.0365
28	Fungi	*Chaetomium globosum*	hg	5692	5647	99.21	–0.223 (± 0.058)	230.690 (± 11.844)	0.98	34336	594	290	137.78	0.0013
29	Fungi	*Coprinopsis cinerea*	or	6143	6138	99.92	–0.176 (± 0.072)	219.845 (± 16.101)	0.92	37500	559	280	54.21	0.0007
30	Fungi	*Phanerochaete chrysosporium*	fc	5688	5646	99.26	–0.265 (± 0.166)	232.617 (± 29.379)	0.9	30000	485	279	17.14	0.0537
31	Protista	*Aureococcus anophagefferens*	a6	7871	7664	97.37	–0.159 (± 0.067)	201.023 (± 10.281)	0.96	32000	543	245	22.34	0.1327
32	Protista	*Dictyostelium discoideum*	dt	6643	6597	99.31	–0.251 (± 0.098)	227.656 (± 20.211)	0.95	34000	–	295	73.82	0.001
33	Protista	*Giardia lamblia*	gf	2426	2348	96.78	–0.119 (± 0.005)	221.790 (± 0.882)	1	1192	630	279	2714.7	0.0122
34	Protista	*Monosiga brevicollis*	ov	5777	5691	98.51	–0.238 (± 0.052)	214.210 (± 10.717)	0.98	38648	–	284	147.68	0.0012
35	Protista	*Naegleria gruberi*	eb	8619	8607	99.86	–0.201 (± 0.129)	216.458 (± 23.734)	0.87	36000	543	268	19.87	0.021
36	Protista	*Paramecium tetraurelia*	ir	15858	15773	99.46	–0.213 (± 0.093)	208.394 (± 17.651)	0.9	200000	550	265	28.29	0.013
37	Protista	*Phaeodactylum tricornutum*	hr	5800	5784	99.72	–0.207 (± 0.095)	211.022 (± 16.193)	0.87	2753	–	255	20.58	0.0201
38	Protista	*Tetrahymena thermophila*	hy	11268	11174	99.17	–0.223 (± 0.120)	228.480 (± 27.268)	0.91	103927	825	303	39.97	0.0032
39	Protista	*Thalassiosira pseudonana*	tl	6238	6230	99.87	–0.184 (± 0.104)	206.013 (± 17.869)	0.86	25000	–	259	24.58	0.0077
40	Protista	*Bigelowiella natans*	bn	490	486	99.18	–0.210 (± 0.084)	207.501 (± 18.090)	0.89	91405.9	337	294	25.29	0.0152
41	Archaea	*Archaeoglobus fulgidus*	af	1573	1571	99.87	–0.239 (± 0.028)	200.756 (± 3.756)	0.96	2178	301	250	65.32	0.004
42	Archaea	*Candidatus Methanoregula*	3p	1549	1548	99.94	–0.245 (± 0.042)	199.534 (± 11.096)	0.94	2542	332	259	115.37	<0.0001
43	Archaea	*Halobacterium salinarum*	8 m	1284	1283	99.92	–0.314 (± 0.056)	213.881 (± 10.952)	0.98	2000	325	262	147.93	0.0012
44	Archaea	*Hyperthermus butylicus*	5 m	983	977	99.39	–0.180 (± 0.031)	197.889 (± 4.878)	0.99	1667	309	238	187.21	0.0464
45	Archaea	*Methanocorpusculum labreanum*	4 l	1128	1121	99.38	–0.304 (± 0.040)	211.796 (± 5.493)	0.92	1804	322	255	21.71	0.0431
46	Archaea	*Natronomonas pharaonis*	np	1553	1552	99.94	–0.291 (± 0.021)	213.048 (± 3.713)	0.97	2595	335	269	173.4	<.0001
47	Archaea	*Picrophilus torridus*	p3	1074	1071	99.72	–0.374 (± 0.177)	232.033 (± 30.236)	0.96	1549	332	273	51.29	0.0189
48	Archaea	*Pyrococcus abyssi*	pb	1229	1226	99.76	–0.226 (± 0.041)	209.505 (± 8.813)	0.96	1765	316	258	78.21	0.003
49	Archaea	*Staphylothermus marinus*	0e	932	932	100	–0.232 (± 0.015)	210.751 (± 1.085)	0.91	1570	324	258	28.65	0.0128
50	Archaea	*Sulfolobus acidocaldarius*	za	1391	1391	100	–0.270 (± 0.043)	221.013 (± 7.876)	0.97	2225	316	267	98.46	0.0022
51	Bacteria	*Acidobacteria bacterium*	a3	3063	3061	99.93	–0.269 (± 0.033)	221.631 (± 12.549)	0.97	5001	384	287	202.13	<0.0001
52	Bacteria	*Cytophaga hutchinsonii*	37	2172	2171	99.95	–0.263 (± 0.010)	217.536 (± 1.671)	0.99	4433	399	279	572.32	<0.0001
53	Bacteria	*Roseiflexus castenholzii*	77	2981	2972	99.7	–0.289 (± 0.104)	229.016 (± 23.424)	0.95	5723	392	289	78.58	0.0009
54	Bacteria	*Leuconostoc mesenteroides*	2 s	1317	1314	99.77	–0.291 (± 0.070)	224.144 (± 13.480)	0.96	2038	337	281	63.78	0.0041
55	Bacteria	*Paracoccus denitrificans*	27	2893	2889	99.86	–0.331 (± 0.182)	226.498 (± 34.921)	0.91	4582	344	278	51.58	0.0008
56	Bacteria	*Polynucleobacter* sp	0 s	1469	1469	100	–0.282 (± 0.055)	222.263 (± 18.110)	0.9	2159	350	286	54.08	0.0003
57	Bacteria	*Syntrophobacter fumaroxidans*	0 l	2674	2674	100	–0.272 (± 0.032)	219.074 (± 8.425)	0.95	4990	376	288	117.56	<0.0001
58	Bacteria	*Arcobacter butzleri*	6 k	1544	1538	99.61	–0.325 (± 0.118)	219.041 (± 22.723)	0.95	2341	354	268	57.99	0.0047
59	Bacteria	*Psychrobacter arcticus*	ri	1447	1442	99.65	–0.246 (± 0.097)	216.765 (± 19.062)	0.9	2650	361	281	36.05	0.0039
60	Bacteria	*Petrotoga mobilis*	6y	1330	1328	99.85	–0.319 (± 0.127)	234.956 (± 26.153)	0.96	2169	361	296	68.67	0.0037

*G.a.* Two-letter genome abbreviation, *L* Average protein length, *L*_*e*_ Effective protein length (sum of domain lengths)

*Missing average protein length information is indicated with a line

Following elaborations by Meyer [28], we consider two levels *i* and *j* of a system to be ‘MA-related’ when (i) the system is hierarchically structured with *n* + 1 levels of organization and *i* > *j* > *n*, (ii) a significant fit of the relation between the length *x* of a higher level *i* sub-system and the average length *y(x)* of the parts of a lower level *j* sub-system exists, and (iii) immediate parts and subsystems (level *i* parts and level *i* + 1 subsystems) are stochastically independent. Specifically, length *x* of subsystem *i* (proteins in proteomes) can be measured by counting terminal (lowest) level *n* parts (amino acids) or by counting the number of level-*j* subsystems (domains). Table 1 therefore shows that domain parts and protein subsystems measured using terminal amino acid parts are MA-related at the proteome system level. We note that the evaluation of 60 proteomes appropriately samples the diversity of the cellular world and meets in every case the fitting requirements of the MA-relationship. It reveals a power law-generating stochastic behavior that is likely universal for proteomes and follows the MA law in a hierarchical system of molecular structure. However, its study only gains empirical interest if a rationale for the MA behavior can be envisioned.

### Menzerath-Altmann’s law links trade-offs between determinants of persistence

Altmann suspected that the MA law was “*somehow connected with the principle of least effort or with some not yet known principle of balance recompensating lengthening on one hand with shortening on the other*” [4]. Here we put forth the hypothesis that the MA law represents a tendency towards economy in a trade-off relationship, where improvement in one property occurs at the expense of others. We will therefore unfold empirical patterns at protein and proteome levels that would support our rationale and mathematical formulations.

In order to interpret the fitting parameters of the MA law in linguistics, a statistical mechanics approach can be used that makes use of classical particle physics to describe words in text [29]. In the absence of a similar approach for protein domain organization, we start by defining a persistence function, which provides a heuristic argument for interpreting the MA power law. We introduce a principle of decreasing returns in domain organization to explain the MA-dependency of Table 1. The principle states that the persistence of a system (*P*) is related to two terms, a cost describing the energy-matter investment in the molecule (*P*_C_) that depends both on *k*, the number of domains in a protein, and *z*_*k*_, the average length of a domain [corresponding to *x* and *y* of eq. (2)], and a term describing the flexibility and robustness of the molecular system (*P*_FR_) that depends on *L*_1_, the length of single domain proteins [i.e., the intercept, which corresponds to *A* of eq. (2); Table 1], *b*, the slope (which describes the decreasing return in domain length *z*_*k*_ with increasing *k*) and *k*. Persistence follows eq. (3)

3 $$ P = {P}_C + {P}_{FR}=-k{z}_k + \frac{L_1}{b+1}{k}^{b+1} $$

The derivative of the persistence function *P* with respect to *k*, when set equal to zero, gives the power law version of the MA formulation [eq. (2)] of eq. (4)

4 $$ {z}_k=A{k}^b $$

with *A* = *L*_*1*_. The function *P* is not always positive; it becomes negative for sufficiently large *k* or *z*_*k*_, beyond the curve *P = 0* in the (*k*, *z*_*k*_) plane. However, eq. [4] corresponds to a ridge of maximum values for *P* between this curve and the *k* and *z*_*k*_ axes. Thus eq. (4) maximizes the persistence function *P*. Substituting eq. (4) into eq. (3), we get along the ridge eq. (5)

5 $$ {P}_{max}=-{L}_1{k}^{b+1} + \frac{L_1}{b+1}{x}^{b+1}={L}_1{k}^{b+1}\left(\frac{1}{b+1}-1\right)=-{L}_1{k}^{b+1}\left(\frac{b}{b+1}\right) $$

Given eqs. (3) and (5), the flexibility plus robustness-to-cost ratio *R* depends on slope *b,* following eq. (6)

6 $$ R=\left|\frac{P_{FR}}{P_C}\right|=\frac{1}{b+1} $$

Steeper slopes (more negative *b*, −1 < *b* < 0) give bigger *R* ratios, which suggest increased trade-offs benefitting flexibility and robustness over economy in the frustrated landscape of molecular persistence. As we will now elaborate, this agrees with *b* representing a measure of structural and functional cooperativity among domains as these accrete in proteins and extend their length.

Multidomain proteins provide both structural and functional plasticity, including an increased repertoire of active, regulatory, allosteric and binding sites, an increased landscape of intramolecular stabilizing interactions, enhanced molecular flexibility, and the option of distributing functions among the different domains [21, 30]. The combination of domains in multidomain proteins by genomic rearrangements, gains and losses manifests quite late in evolution [13, 20], suggesting that domain accretion in proteins is a derived evolutionary trait that benefits the increasing tasks of evolving multi-level molecular and cellular organization. Domains stabilize proteins in multidomain proteins mainly through interaction between hydrophobic residues in inter-domain interfaces [19]. The energy of these interactions scales linearly with the surface area of domain-domain interfaces, which depends on the size of the protein. Interactions also enhance the stability of individual domains, which constrains mutational substitution of interacting residues. This matches the broad observation that surface residues are less conserved in proteins when compared to those that are buried in the structural core (e.g., [31]). A recent comparison of number of buried residues normalized to the radius of gyration of domain structure has shown that younger domains tend to have higher surface area to volume ratio than older counterparts [27]. Since in general, younger domains engage in massive domain combinatorics [13], then multidomain proteins must be enriched in domains with relatively more stable structural cores. Thus, increases in *k* must result in increases of domain cooperativity during folding and consequent increases of protein stability.

If the proteome imparts limits to cellular behavior, then a number of crucial biophysical properties of proteins could constrain proteomic and cellular make up. Biophysical considerations have established that many properties of single-domain proteins, including folding rate and collapse, protein stability and size, and diffusion coefficients, simply depend on chain length and are important for the growth and fitness of the cell [32–35]. Scaling and distribution relationships reveal that folding rate, collapse, size, stability and diffusion of proteins depend simply on chain length [33]. While proteomes were marginally stable to denaturation, the function of cells appeared rate-limited not only by protein synthesis but also by the diffusional transport of proteins (which could explain compartmentalization in eukaryotic organisms) and the folding kinetics of the slowest-folding proteins of the cells. The dependence of cellular processes on protein folding and length is not a surprise. Length is a fundamental biophysical property of biopolymers as they self-assemble to maximize thermodynamic dissipation of energy [35]. Proteins transition abruptly into the folded state through a remarkable cooperative and frustrated process. Hydrophobic residues are buried to form the globular core and charged and polar residues that extend protein structure are exposed. This process exhibits remarkable universal behavior. Folding rates of both proteins and RNA scale as *e*^√*L*^, with *L* representing the length of the polymer. Similarly, the folding and collapse transitions, which coincide, exhibit a cooperative behavior *Ω* that scales with *L*^1.22^ [35]. Therefore, folding cooperativity scales with protein length and therefore with *k* in multidomain proteins.

We reiterate that the persistence function *P* for proteins and proteomes of eq. (3) depends solely on the length and number of domains, and can be apportioned into two separate terms. The first term reflects the matter-energy cost of lengthening domains by addition of amino acids or lengthening proteins by domain accretion. This cost is mainly imposed by protein synthesis, diffusion and folding and delimited by the mass-energy equivalence imparted by biochemistry. For example, shorter proteins that retain maximum rates of function and have similar kinetic characteristics incur in lower metabolic costs of translation [36], as long as the trade-off maximizes cell physiology and growth rates. We note however that the intensity of protein length reductive pressure decreases if the fraction of cellular mass of the protein decreases. This would be particularly significant for highly diverse proteomes (e.g., Eukarya) and macromolecular crowding environments that maximize diffusion rates and the kinetic efficiencies of proteins [25]. Similarly, domain length follows a narrow distribution [37], limited by the benefits of fast folding of shorter proteins and the stability offered by burial of hydrophobic residues of structural cores of sufficient size. The second term of *P* reflects the benefits of larger domains and multidomain proteins, which contribute intramolecular interactions and provide additional structural and functional bases for increasing information flux through the system and enhancing flexibility and robustness. Borrowing from Yafremava et al. [38], we here define flexibility broadly as those structural and functional mechanisms that respond to changes internal and external to the molecular system and require processing of information. More flexible systems are generally larger, harbor more complex functionalities, and are more diverse in finding trade-off solutions. We define robustness as mechanisms that use information to maintain structure and function despite external influence and protect molecules from malfunction. Robustness includes stability but refers to broader processes that are passive from an information point of view. Information in molecules is stored in intramolecular and intermolecular interactions necessary for molecular function and stability [39]. In domain combinations, information also materializes in the combinatorics of domains, which manifests at chain and 3-D levels, and can be equated with language information [21].

**Fig. 2:**
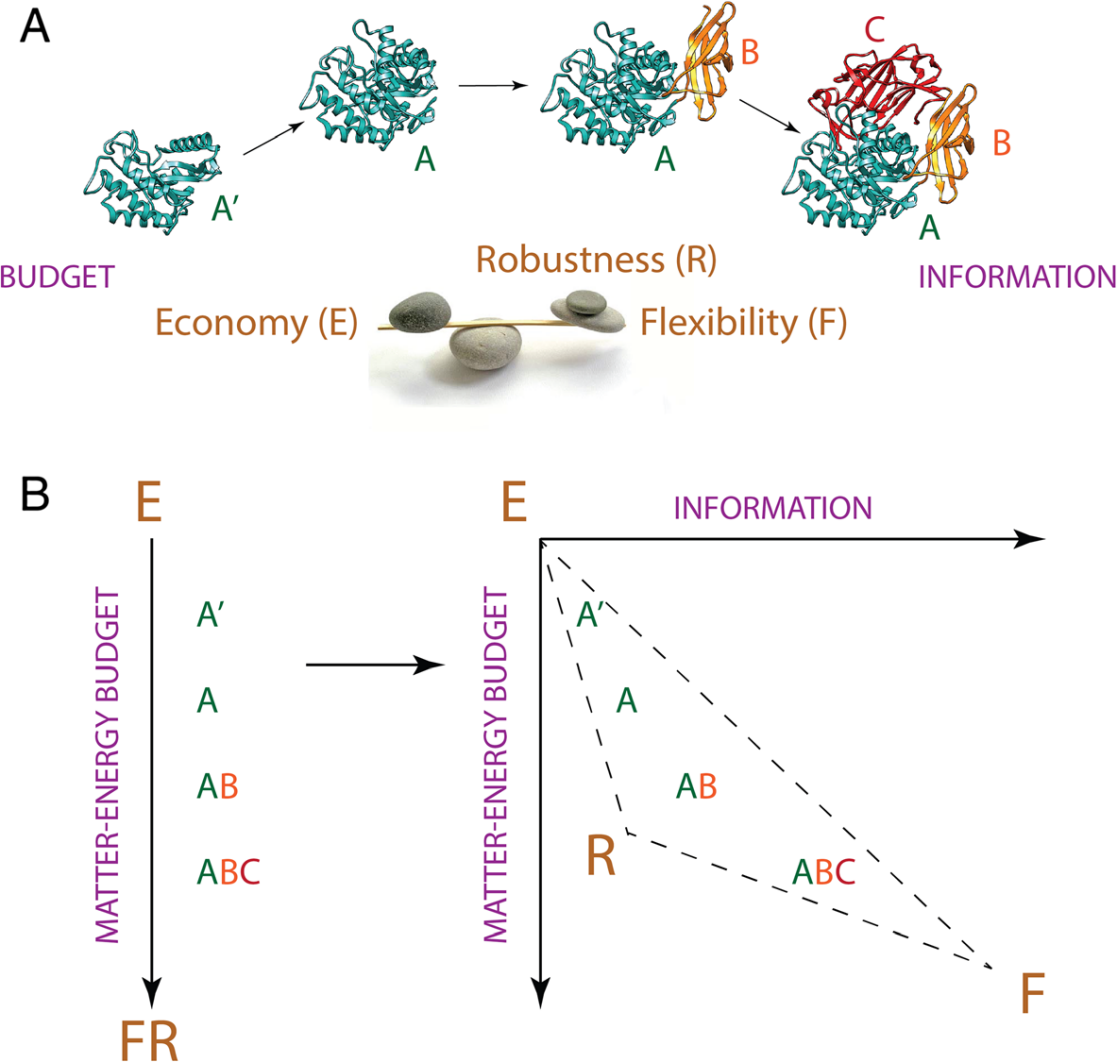
Domain size and organization affects molecular persistence. **a** Proteins with different propensities towards economy (E), flexibility (F) or robustness (R) will find optimal trade-off solutions given matter-energy budget and information. **b** Molecules segregate along a budgetary axis (*left panel*) in the order A’, A, AB and ABC, where letters indicate their domain makeup. The length of A’ is smaller than that of A. Larger proteins are more expensive to make and maintain but provide flexibility and robustness benefits. Once an information flux is made explicit (*right panel*) the segregation transforms into a triangle that unfolds trade-off relationships between flexibility and robustness. The extended vertex of flexibility is mainly driven by new levels of structural organization, such as the combination of domains in proteins, formation of quaternary structures and emergence of protein complexes. These levels impose additional constraints on economy that are always satisfied by the MA law at different levels of the hierarchy

The persistence function therefore makes a mathematically explicit framework of persistence strategies for biomolecular systems, in which economy, flexibility and robustness engage in various trade-off solutions. This framework defines a ‘triangle of persistence’, which has the potential to successfully explain organismal diversity [38]. Figure 2 summarizes the framework as it applies to domain structure and organization.

### Patterns of decreasing returns in proteomes of kingdoms and superkingdoms

**Fig. 3:**
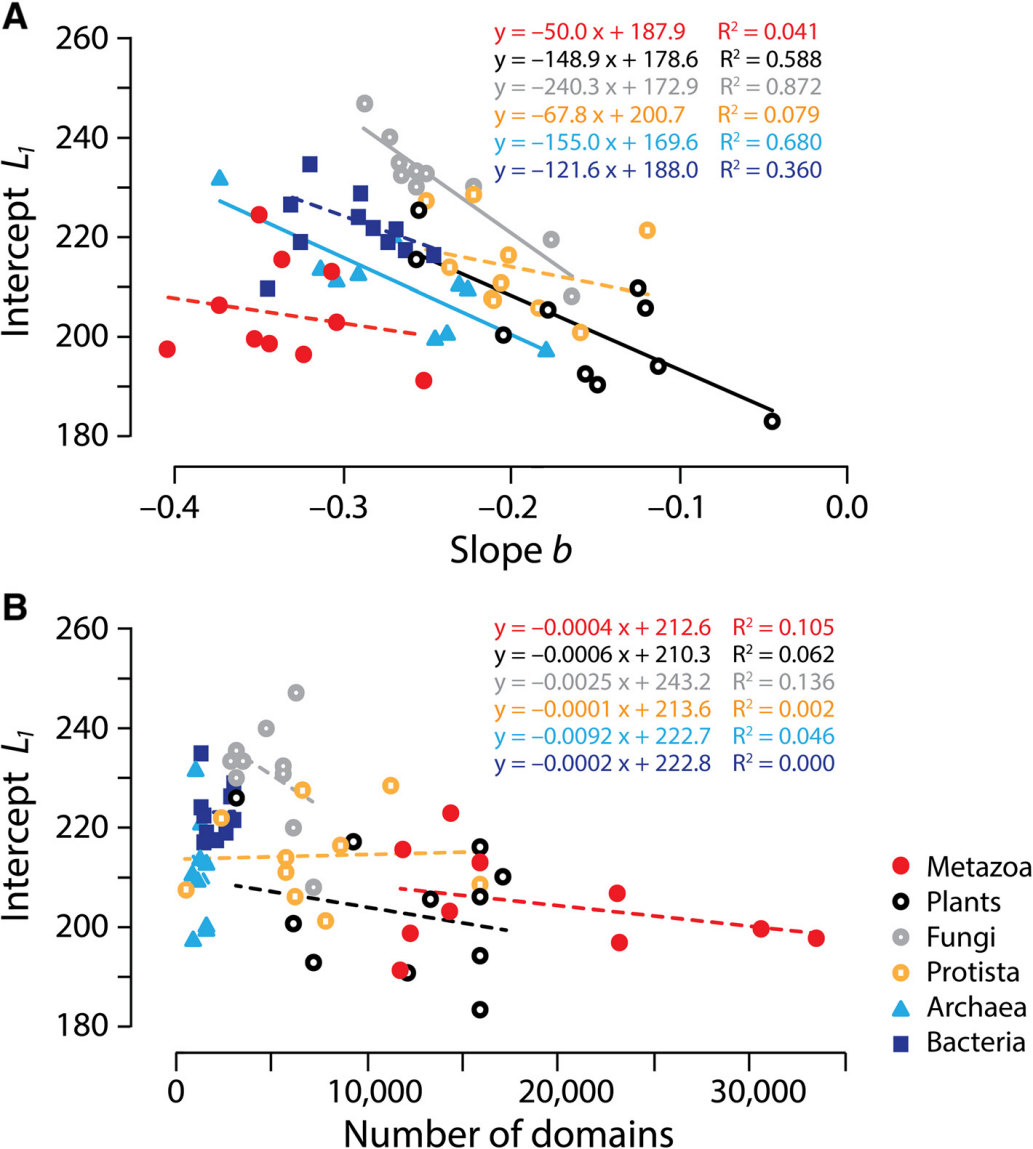
Patterns of decreasing returns in proteomes of Kingdoms and Superkingdoms. **a** Slope *b* vs. intercept *L*_*1*_ plot. Slope and coefficients of determination (R^2^) values for correlations in organismal groups are shown for each fitted line. Most fungi exhibited the largest *L*_*1*_ intercepts were largest for most fungi and smallest for a substantial number of plants and metazoans. Most metazoans exhibited the steepest *b* slopes and most plants and protists the shallowest. **b** Total number of domains vs. intercept *L*_*1*_ plot. The total number of domains is the total count of protein domains in proteome analyzed. Dashed trend lines described non-significant fits (*p* > 0.05)

The MA-law imposes patterns of decreasing returns for domain lengths of proteins of a proteome. These patterns relate to protein domain make up, domain function, and evolutionary pressures imposed on the proteome as an interacting body of the cell. Analyses of domain length in proteins sampled from many proteomes (e.g., a set of PDB structures [37]) may not reveal the MA relationship because the scaling patterns are global and proteome centric. Conversely, a simple comparative analysis of the complement of protein domains in four kingdoms of Eukarya and superkingdoms Archaea and Bacteria hold very distinctive distributions of molecular functions [40] and domain rearrangements [20]. Thus, it is expected that specific patterns of decreasing returns will exist for those groups. We therefore plotted slope (*b*) versus intercept (*L*_1_) for each proteome that we studied with the goal of dissecting the contributions of economy and length of domains in single domain proteins that are characteristics of organismal groups (Fig. 3a). The lengths of single domain proteins *L*_1_ act as upper bounds for the MA’s ‘shortening’ principle of domain length, establishing a flexibility-robustness stratum for a proteome in the triangle of persistence. Slopes ranged from −0.045 for *Medicago truncatula* (Plants) to −0.404 for *Brachiostoma floridedae* (Metazoa). Intercepts ranged from 183 for *Medicago truncatula* to 247 for *Aspergillum nidulans* (Fungi). Most fungi exhibited the largest intercepts and a substantial number of plants and metazoans showed the smallest. Higher intercepts should be interpreted as larger ‘starting’ domain sizes fostering opportunities for flexibility and robustness but counteracted by increased burdens of cost. Most metazoans showed the steepest slopes and substantial number of plants and protists the shallowest. Steepest slopes should be interpreted as stronger ‘push’ towards flexibility and robustness and corresponding ‘counter-push’ towards economy in domain organization. Proteomes distributed in the plot following a fan-like pattern, with the top segment of the semi-circle occupied by Fungi, Protista-Bacteria-Plants, and Archaea, in that order, and the bottom part by Metazoa. Plants and Protista occupied the fan handle.

We find that proteomes in the plot showed higher linear correlations for Fungi, Archaea and Plants (R^2^ = 0.59-0.87; *F* = 11.4-54.6; *p* < 0.0001-0.01), the lowest correlation for Bacteria (R^2^ = 0.36; *F* = 4.51; *p* = 0.067), and no significant trends for Metazoa and Protista (R^2^ = 0.04-0.08; *F* = 0.34−0.68; *p* = 0.432-0.575). Since slopes of proteome groups in the slope *b* versus intercept *L*_1_ plots increase with single-domain length (intercept *L*_1_) and increasing linear fits, we hypothesize that this increasing trend, which is maximal in Fungi, describes a ‘compressible’ property capable of reducing domain length (*L*_*k*_) when additional domains are accreted in proteins (*k* > 1). In other words, proteomes like those of fungi that exhibit on average longer domains in single domain proteins are capable of considerable length reduction as domains accrete in proteins. In turn, those that have shorter average single domain proteins relax the reductive tendency in multidomain proteins. Given the theoretical link that exists between *b* and both domain cooperativity and stability elaborated above, and the high surface area to volume ratio detected in new emergent proteins [27], we propose that the ‘compressible’ property is associated with contact density in domain structures, i.e., the fraction of buried sites in the atomic structure. Contact density correlates positively with evolutionary rate, measured as substitutions in protein sequence, without being confounded by gene expression levels [41]. Consequently, the larger numbers of contacts buried in the structures of larger domains, such as those of fungi, are prone to increased structural change. This could accelerate the reduction of the length of secondary structures by domain accretion in multidomain proteins, as accretion increases buried surface area. Since domains in a multidomain protein are translated at the same rate, the effect of gene expression levels on sequence change homogenizes differences in evolutionary rates of domains in multidomain proteins [42]. Thus, increases in evolutionary rates with domain number should extend to the entire protein. We note that both fungi and plants, as a group, are subject to increased levels of genomic rearrangements (via high recombination rates or transposon activities), when compared to metazoan, bacterial and archaeal microbes. This could result in increased insertion-deletion (indel) dynamics in regions of secondary structure that would decrease the length of these segments in evolution. Moreover, organismal groups such as Archaea and Fungi are subjected to strong reductive evolutionary pressures [43] that manifest in highly reduced proteins and proteomes [25]. This trend adds ‘compression’ tendencies to the length of multidomain proteins in this group, even if the lengths of single domain proteins are on average low.

We also plotted total number of domains in proteomes versus intercept (*L*_1_) to reveal the effect of reductive evolution at proteome level on starting domain size of organismal groups (Fig. 3b). As expected, the proteomes of the microbial superkingdoms were highly reduced, an evolutionary tendency imposed by an early pressure of demanding microbial lifestyles to reduce protein complements [38, 43]. However, proteomes of Bacteria showed larger *L*_1_ values than those of Archaea, uncovering additional reductive evolutionary constraints imposed on the archaeal microbes by lifestyle and history. With exception of Fungi, the rest of eukaryotic kingdoms relaxed reductive evolutionary constraints. Metazoa showed the largest repertoires and low *L*_1_ domain lengths. Fungi showed the smallest repertoires and the largest *L*_1_ values. All organismal groups in the plot were clearly dissected but none showed significant correlations (*R*^*2*^ = 0.001-0.136).

### Patterns of domain length over-representation in single domain proteins

**Fig. 4:**
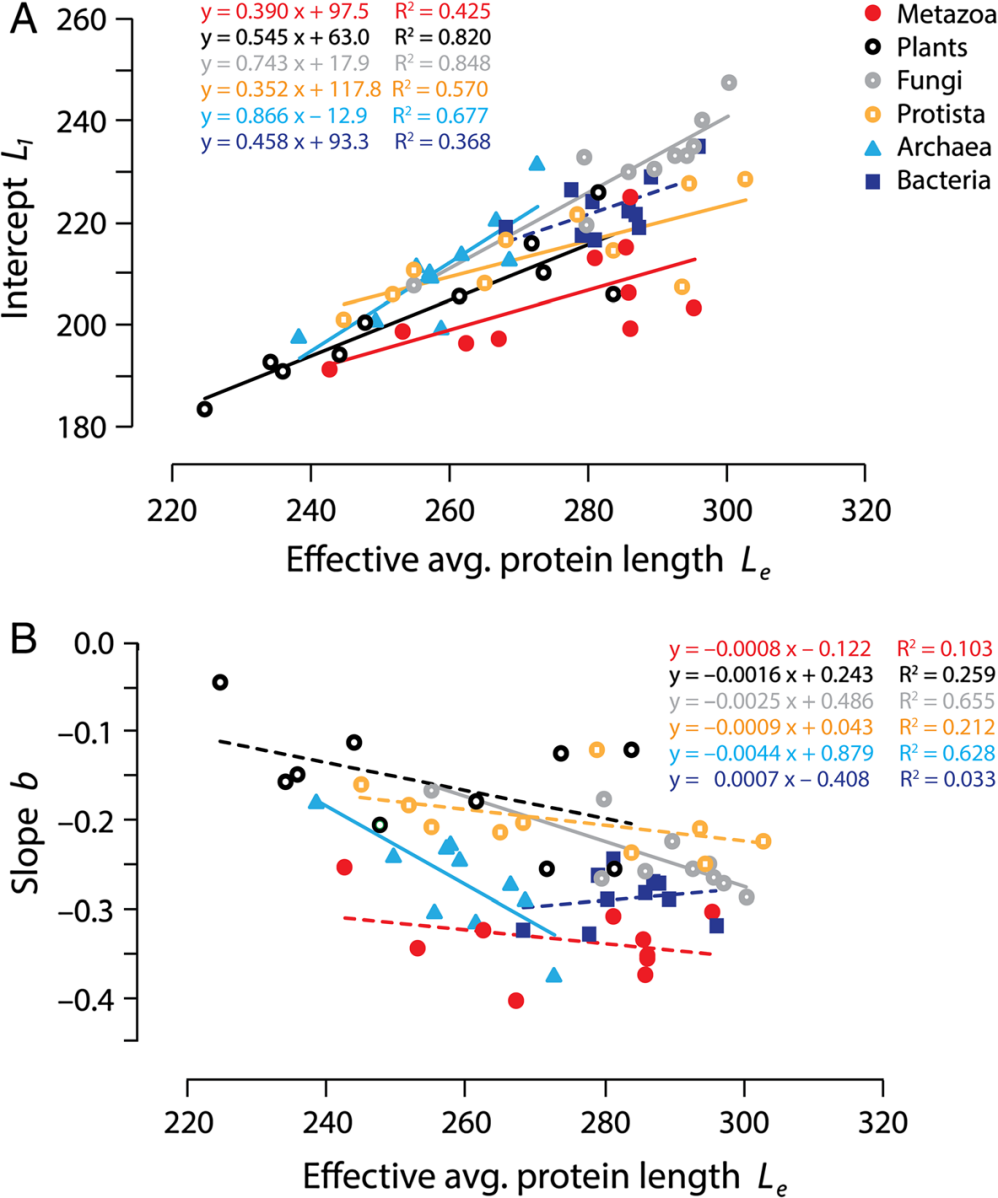
Patterns of domain length overrepresentation in single domain proteins. **a** Intercept *L*_*1*_ vs. effective average protein length *L*_*e*_ plot. *L*_*e*_ represents the sum of the length of individual domain constituents of a protein, without considering linkers and terminal non-domain sequences. *L*_*e*_ were calculated using weights *M*_*k*_, the number of proteins with *k* domains, and averaging over all *k* up to *K’*, the largest value of *k* on the linear part of the log-log plot (see Fig. 1 as example). **b** Effective average protein length *L*_*e*_ vs. slope *b* plot. Slope and coefficients of determination (R^2^) values for correlations in organismal groups are shown for each fitted line. Dashed trend lines described non-significant fits (*p* > 0.05)

The effective average protein length (*L*_*e*_) represents the sum of the length of individual domain constituents of a protein, without considering linkers and terminal non-domain sequences. We calculated *L*_*e*_ for each proteome using weights *M*_*k*_, the number of proteins with *k* domains, and averaging over all *k* up to *K’*, the largest value of *k* on the linear part of the log-log plot. The plot *L*_*1*_ versus *L*_*e*_ (Fig. 4a) showed linear correlations with low goodness-of-fit for proteomes in all kingdoms and superkingdoms (*R*^*2*^ = 0.42-0.85; *F* = 5.92-44.74; *p* = 0.0002-0.041) with the exception of Bacteria (*R*^*2*^ = 0.37; *F* = 4.66; *p* = 0.063). All trend lines clustered together quite tightly showing an expected overall increase of *L*_*1*_ with increasing *L*_*e*_. The slopes, which vary from 0.352 to 0.866, represent the fraction of total domain length apportioned to single domain proteins (*L*_*1*_/*L*_*e*_). Slopes show the disproportionate large representation of single domain proteins in microbial proteomes that hold only a limited repertoire of multidomain proteins. Slopes are maximal in Fungi and Archaea (0.866 and 0.742), intermediate in Plants and Bacteria (0.545 and 0.458) and minimal in Protista and Metazoa (0.352 and 0.390). Thus, Fungi and Archaea have significant overrepresentation of the length of single domain proteins, a feature that correlates with the high ‘compressible’ property revealed in Fig. 3a and the fact that they represent the organismal groups subjected to highest reductive tendencies in microbial and eukaryotic superkingdoms, respectively, revealed in Fig. 3b. The steepness of slopes follows the Fungi–Archaea > Plants–Bacteria > Protista–Metazoa trend of the slope versus intercept plot. Similarly, the best supported linear fits correlate with proteomes harboring larger proteins resulting from larger single domain proteins. Archaea is the superkingdom harboring the most reduced protein domain repertoires and the shortest proteins [25, 43]. This reductive trend is likely the result of mass economy and growth rate optimization. It is therefore unsurprising that it is costly for archaeal proteins to add more domains to a single domain protein; *L*_*1*_ takes more of *L*_*e*_. A similar trend exists in fungi, especially in ascomycetous yeast, which already show significant reductive trends compared to other fungi and other eukaryotes [40] (Nasir, A. and Caetano-Anollés, unpublished). In our study, ascomycetes that include unicellular yeasts and dimorphic fungi that switch between unicellular and hyphal phases, have on average higher *L*_*1*_ (236 ± 6) and steeper slopes (−0.259 ± 0.018) than the rest of fungi examined (220 ± 10 and −0.202 ± 0.045), supporting the reductive trend visible in Fig. 3b. Within Eukarya, fungi also show maximum reductive evolutionary tendencies in the repertoire of domains and associated functions, when these are defined at fold superfamily level of structural classification (see Table S1 in [40]).

We also plotted *L*_*e*_ versus slope *b* again revealing linear correlations for Fungi and Archaea with low goodness-of-fit (*R*^*2*^ = 0.63-0.66; *F* = 13.48-15.22; *p* = 0.0045-0.0063) but non-significant fits for the rest (Fig. 4b). Most correlations showed that *b* became steeper with increasing *L*_*e*_. This is expected since larger proteins must impose increased pressure to fulfill the decreasing return strategy of the MA law and the principle of maximum economy. Remarkably, groups showing the more significant linear correlations (Fungi and Archaea) showed maximum slopes in the plot, matching patterns observed in Fig. 3a. Thus, the marked reductive evolutionary trends of Archaea and Fungi that manifest at proteome level carry over to the length of individual proteins, supporting a previous study of reductive evolution [25]. We note that in Fig. 4b, the slope of the archaeal group is steeper (−0.0044) than that of fungi (−0.0025), revealing additional reductive constraints that are imposed on the akaryotic microbial superkingdom, which is significantly marked and unfolded very early in protein evolution [43]. This is also evident in the plot of Fig. 3b.

## Conclusions

Processes of diminishing returns manifest when systems search for optimality. The closer to the optimum condition, the more difficult the effort invested in attaining it. For example, laboratory optimization of an arylesterase function in an *in vitro* evolution experiment revealed strong diminishing returns on enzymatic activity [44]. The first mutations in the bacterial population accounted for most improvements and the last ones simply reinforced the effects of early ones. In general, experiments that unfold new molecular functions also reveal the existence of evolutionary trade-offs between stability and function (e.g., [45]). Here we uncover similar processes of diminishing returns and trade-offs operating during molecular accretion of domains in proteins.

Menzerath’s insight suggested the existence of a universal tendency of parts to decrease their size when systems enlarge. The MA law appears universal for language and music. Our study extends its validity to biological parts and systems. In language, constituents of language constructs, such as the phonemes of words, are dynamic. They change as language unfolds in human history. Similarly, parts of biological systems, such as the domains of proteins, change in molecular evolution. In the case of domains, they increase or decrease in length and accrete in multidomain proteins by the pervasive effects of mutations and genomic rearrangements. We now find that protein domain length decreases with increasing number of domains in the proteins of proteomes. The existence of an MA law in protein domain organization can be explained as the consequence of the frustrated interaction between the strategies of economy, flexibility and robustness. The MA law represents a power law relationship that manifests when unfolding molecular persistence *P* as a function of domain accretion, measured as number of domains *k* in proteins. *P* holds two terms, one reflecting the matter-energy cost of adding domains and extending their length in proteins, the other reflecting how domain length and number impinges on information and the flexibility and robustness of the molecular system. Thus, our persistence function describes a frustrated landscape in a ‘persistence triangle’ with vertices representing the three main strategies.

A previous analysis of proteome makeup revealed that organisms in kingdoms and superkingdoms preferentially use flexibility and robustness properties in trade-off relationships with economy as they face environmental uncertainties and negotiate survival [38]. Archaea and the more flexible Bacteria gravitate towards the triangle’s economy vertex. In turn, eukaryotic organisms trade economy for flexibility and robustness as they massively expand biological repertoires and levels of organization. Protista occupy a saddle manifold separating Archaea and Bacteria from multicellular organisms. Plants and the more flexible Fungi are less affected by the positive feedback loop that pushes Metazoa towards maximum flexibility. Our mathematical formulations of persistence, which explain the MA power law, manifest similar trade-off relationships in the proteins of proteomes (Figs. 3b and 4b). Archaea, Fungi and to a lesser degree Plants show the largest push towards economy, each at their economic stratum. Fungi increase domain size in single domain proteins while reinforcing the pattern of diminishing returns in multidomain proteins. Archaea and Plants follow the same strategy but relaxing the push towards larger single domain size. In contrast, Metazoa, and to lesser degrees Protista and Bacteria, relax the MA pattern of economy returns within a broad range of single domain sizes. Metazoa achieves maximum flexibility and robustness in proteins by generating compact molecules with a large number of domains and a multiplicity of combinations. This strategy implemented by Metazoa offers a new vocabulary for molecular functions in biology and new levels of structural organization.

## Methods

We selected 60 proteomes of free-living species from the highly curated dataset of Wang et al. [25], which holds ~ 3 million sequences (from 745 proteomes) with structural domains assigned using hidden Markov models (HMMs) of structural recognition in SUPERFAMILY [46]. Species covered superkingdoms Archaea and Bacteria and the four main kingdoms of Eukarya, Protista, Plants, Fungi and Metazoa (animals). Protein entries were retrieved trusting the reliability and robustness of HMMs that were used to delimit domains, the low probability of cryptic domains matching non-domain linker sequences (*P* < 0.0001) that could affect assignments of sequences to multi-domain protein groups, and the absence of biases imposed on length estimates by superkingdom-specific Markovian models [25]. A flat file was created with information about protein ID, domain ID defined at superfamily level, domain length and whole protein length. We averaged out domain lengths (*Y*_*k*_^*j*^) against each domain number (*k*) for the selected proteins. The following eqs. (7) and (8) were then used to calculate the mean value (*z*_*k*_) and variance (*s*_*k*_^*2*^) respectively.

7 $$ {z}_k = \frac{{\displaystyle {\sum}_{i=1}^{M_k}}{Y}_k^j}{M_k} $$

8 $$ {\left({s}_k\right)}^2=\frac{{\displaystyle {\sum}_{j=1}^{M_k}}{\left({Y}_k^j-{z}_k\right)}^2}{M_k-1} $$

where z_k_ = mean value of Y_k_^j^ within a *k*, *Y*_*k*_^*j*^ = sum of the value for *M*_*k*_’s at k point, *M*_*k*_ = number of proteins with *k* domains, *i* = number of unique domains starting from 1 to *M*, *k* = unique domain number, *j* = number of *Y*_*k*_ points starting from 1, and *(s*_*k*_*)*^*2*^ = variance.

The graphs of *k* versus z_k_ were plotted with both axes on a log_10_ scale. To avoid biases introduced by a small minority of proteins harboring a large number of domains (outliers with *k* ≤ *K* domains), we excluded proteins with more than *K’* domains and used the rest to fit the lines. *K’* was chosen by eye with the goal of maximizing both R^2^ and the number of proteins retained. Initial boundaries for the optimization were *R*^*2*^ > 0.8 and > 95 % of protein entries retained. Analysis of several proteomes in preliminary studies showed that the by-eye choice of *K’* judged by marked departures from a line gives nearly optimal fit. For example, inclusion of proteins with *K’* ≥ 14 domains of *H. sapiens* in the example of Fig. 1 (up to the maximum of 20) decreases the R^2^ statistics from 0.91 to 0.7. In turn, selecting *K’* ≤ 5 domains decreases the number of proteins retained from 99.7 to 95 %. This brackets the *K’* = 13 domain boundary by exactly *k* = ±7.

Lines were fitted in log space to eq. (9)

9 $$ {z}_k=A{k}^b $$

using the Excel solver for weighted and non-weighted least squares of Harris [47], which fits experimental data using non-linear functions. For the solver input, we used *k (k = 1 to K’), z*_*k*_, standard errors of the means (*Y*_*err*_), and weight of *k*th value (*w*_*k*_) to calculate the slope (*b*), intercept (*L*_*1*_) and their respective standard errors of the means (SEM). We used the following eqs. (10) and (11) to calculate (*Y*_*err*_) and (*w*_*k*_):

10 $$ {Y}_{err}=\kern0.5em \sqrt{\frac{{\left({s}_k\right)}^2}{M_k}} $$

11 $$ {w}_k = \frac{M_k}{{\left({s}_k\right)}^2} $$

Effective average protein lengths (*Le*) were calculated using the following eq. (12)

12 $$ {L}_e=\frac{{\displaystyle {\sum}_{k=1}^{K\hbox{'}}}\left(k*{M}_k*{z}_k\right)}{{\displaystyle {\sum}_{k=1}^{K\hbox{'}}}{M}_k} $$

We used the *F* statistics of Proc GLM (SAS, SAS Inst. Inc., Cary, NC) to test the linear relationship between *k* vs. *z*_*k*_, *b* vs. *L*_*1*_, genome size vs. *L*_*1*_, *L*_*e*_ vs. *b* and *L*_*e*_ vs. *L*_*1*_. We report dependencies that are most useful for biological interpretation. In particular, *L*_*1*_ describes the average length of single domain proteins, which serves to define an upper bound for the MA-dependency of a proteome. In turn, *L*_*e*_ describes the sum of the length of individual domain constituents of a protein, which is an indicator of mass economy for growth rate optimization. An example of a regression model is given by eq. (13)

13 $$ {V}_{ij}={L}_1+b{U}_i + {\varepsilon}_{ij} $$

where V_*ij*_ is the observation of the *i*th effect and the *j*th replication, *U*_*i*_ is the *i*th effect, and *ε*_*ij*_ is a random error term of the *i*th effect and *j*th replication, assuming NID (0, σ^2^), i.e., normality, independence and identical data distribution.

### Availability of supporting data

A file with the proteomic data of Wang et al. [25] analyzed in this study can be found at LabArchives: http://dx.doi.org/10.6070/H4513W6X.

## Abbreviations

HMMs: Hidden Markov models
MA: Menzerath-Altmann
